# Prolonged *In Vivo* Performance of Electrospun PCL-Based Vascular Grafts in a Large Animal Model: Influence of Material Design and Systemic Drug Support

**DOI:** 10.1021/acsomega.6c00128

**Published:** 2026-03-13

**Authors:** Suzan Ozdemir, Janset Oztemur-Sezgin, Havva Tezcan-Unlu, Atıf Yolgosteren, Elif Unlu, Abdullah Dogukan Oz, Meric Kocaturk-Guney, Gulsah Cecener, Nihal Yasar Gul Satar, Hande Sezgin, Ipek Yalcin-Enis

**Affiliations:** † Textile Engineering Department, Textile Technologies and Design Faculty, 52971 Istanbul Technical University, Istanbul 34437, Türkiye; ‡ Department of Medical Biology, Faculty of Medicine, 37523 Bursa Uludag University, Bursa 16059, Türkiye; § Department of Cardiovascular Surgery, Faculty of Medicine, Bursa Uludag University, Bursa 16059, Türkiye; ∥ Department of Surgery, Faculty of Veterinary Medicine, Dokuz Eylül University, İzmir 35400, Türkiye; ⊥ Department of Veterinary Internal Medicine, Faculty of Veterinary Medicine, Bursa Uludag University, Bursa 16059, Türkiye; # Department of Surgery, Faculty of Veterinary Medicine, Bursa Uludag University, Bursa 16059, Türkiye

## Abstract

The development of biodegradable vascular grafts that replicate the structural and functional characteristics of native vessels remains a critical challenge in regenerative medicine. Although *in vitro* studies have demonstrated promising outcomes, the long-term success of electrospun vascular grafts in large animal models has been limited due to mechanical incompatibility, insufficient interfacial adhesion, and thrombotic issues. In this study, bilayer vascular grafts composed of polycaprolactone (PCL), polylactic acid (PLA), and poly­(l-lactide-*co*-caprolactone) (PLCL) were developed and fabricated using the electrospinning technique, and after testing biological and mechanical performance, they were implanted and evaluated in a porcine carotid artery model. Two bilayer configurations were designed, namely, PCL_PCL and PCLPLA_PLCL, consisting of random fiber distribution in inner layers and radial fiber orientation in outer layers to mimic native arteries. Mechanical test results revealed that developed grafts provided adequate performance in terms of tensile strength, burst pressure, compliance, and suture retention strength when compared with native carotid arteries. PCLPLA_PLCL grafts exhibited higher radial strength due to the presence of PLA but showed lower compliance. *In vivo* results indicated early graft failure in PCLPLA_PLCL samples, which might be associated with delamination at the interface. In contrast, PCL_PCL grafts achieved longer patency, particularly in animals receiving enoxaparin via intradermal injection, with one graft remaining partially patent for up to 90 days, a finding rarely reported in porcine studies. Histological evaluation revealed that inflammation, fibrin deposition, and fibrotic tissue formation were the primary causes of graft occlusion. Overall, the results demonstrated that material selection and pharmacological management play decisive roles in the long-term performance of small-diameter vascular grafts. Electrospun PCL-based bilayer constructs appear to hold significant potential for future clinical applications.

## Graphical abstract

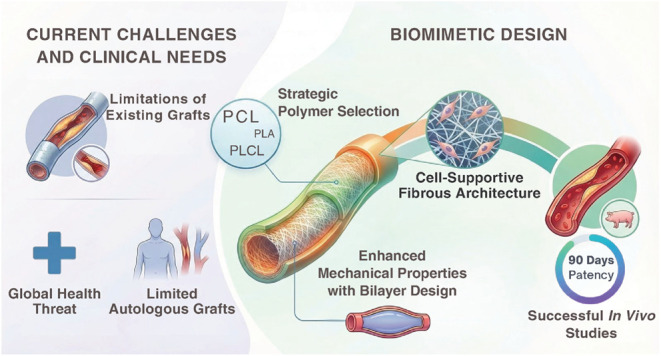

## Introduction

Vascular replacement and bypass remain the most widely employed surgical interventions worldwide for treating arteries affected by atherosclerosis, infection, or severe trauma.^1^ Various xenograft transplantation methods have been tested in humans, but none have been approved for widespread clinical use due to immune responses and the requirement for long-term immunosuppressants. Consequently, polymeric materials are the preferred choice for large vessel graft replacements (over 6 mm), with Dacron and ePTFE grafts being the most commonly used materials.^2^ This kind of graft has a high success rate of up to 90% for large-diameter replacements.^3,4^ However, these grafts are prone to thrombosis in small-diameter vessels due to slow endothelialization and blood protein adhesion, leading to clot formation without an endothelial layer, besides pseudoaneurysm, occlusion from myointimal hyperplasia, and congenital heart failure. Additionally, infection and neointimal hyperplasia, caused by increased bacterial sensitivity to foreign materials, limit their use in small-diameter vessels (under 6 mm).^5–7^ Therefore, there is a growing clinical demand for vascular grafts that accurately replicate the complex structure, anatomy, physiology, and biomechanics of human blood vessels.^7,8^ These grafts should serve as replacements for native vessels in treating cardiovascular disorders, ideally possessing sufficient mechanical strength to endure blood pressure and surrounding tissue stresses while promoting cellular activity during biodegradation to ensure native vascular wall regeneration and prevent inflammation.^9^

There are numerous vascular graft constructions currently in use or under development, ranging from traditional textile-based structures, such as woven and knitted fabrics, to advanced nano- and microfibrous surfaces produced by modern fabrication techniques. Although woven structures are widely used in commercial vascular grafts due to their mechanical robustness and dimensional stability, their low porosity, limited compliance, and difficulty in suturing restrict their application to high-pressure regions such as the thoracic aorta.^10^ Knitted grafts, by nature of their looped structure, tend to be more elastic and porous than woven ones. These features can ease surgical manipulation and promote tissue integration, yet they also introduce drawbacks. The relatively large pore size often leads to blood leakage, necessitating additional processing steps such as precoagulation and crimping.^11,12^ However, such modifications are not without consequence, as they may elevate the risk of thrombosis in small-caliber applications, limiting the clinical use of knitted grafts to larger arteries.^13^ In this context, these fabrication methods fall short of replicating the structure of the extracellular matrix (ECM), which is crucial for tissue-engineered vascular grafts designed for neo-vessel regeneration.^14^ However, fibers produced via electrospinning mimic the natural three-dimensional microenvironment of the ECM. This technique enables the precise control of fiber morphology, diameter, orientation, porosity, pore size, and wall thickness, as well as the creation of multilayered designs that effectively replicate the layers of native blood vessels, including the *tunica intima*, *media*, and *adventitia*.^15^ The choice of biomaterial plays a crucial role as construction selection in ensuring clinical success for tissue-engineered vascular grafts. These materials must be biocompatible to avoid immune responses and should degrade at a rate that matches tissue regeneration. They are also expected to replicate the mechanical behavior of native vessels, particularly elasticity and tensile strength, to withstand physiological loads. Equally important is their ability to mimic the ECM, which promotes cell adhesion, proliferation, and differentiation, thereby facilitating proper endothelialization and reducing the risk of thrombosis.^16^ In addition to these biological and mechanical criteria, materials must be sterilizable and suitable for scalable manufacturing.^17^ Natural polymers, such as collagen, gelatin, elastin, chitosan, and silk fibroin, are frequently used due to their biocompatibility and ability to support cell-related processes. In contrast, synthetic polymers like polycaprolactone (PCL), polylactic acid (PLA), poly­(l-lactide-*co*-caprolactone) (PLCL), polyglycolic acid (PGA), and poly­(lactide-*co*-glycolide) (PLGA) are favored for their mechanical strength and tunable degradation profiles, which make them well-suited for constructing durable grafts in regenerative applications.^18,19^

On the other hand, the search for an ideal vascular graft design has been ongoing for decades, driven by the limitations of currently available options. In this context, numerous *in vitro* studies have been reported in the literature, demonstrating promising results.^1,20–24^ However, the dynamic environment of a living body may not always align with these study outcomes. In this context, *in vivo* studies are crucial, offering critical clues for the development of an ideal graft. Zhao et al. reported successful long-term patency in rats using PCL/fibrin (80/20) vascular grafts with perioperative aspirin administration. After 9 months, no thrombosis or platelet aggregation was observed, and a continuous endothelial monolayer resembling native arteries was detected within the graft lumen.^25^ Furthermore, Dokuchaeva et al. implanted PCL-based electrospun grafts into the abdominal aorta of rats and applied postoperative Fraxiparin. Histological analysis revealed progressive cellular infiltration up to day 60, followed by reduced cellularity at day 90, likely due to accumulated polymer degradation products despite preserved graft architecture. By day 60, a continuous endothelial lining with signs of intimal hyperplasia was observed.^26^ On the other hand, Fang et al. and Xiao et al. both evaluated bilayered electrospun grafts in rabbit models with systemic anticoagulation (heparin and enoxaparin, respectively). Fang used thermoplastic polyurethane (TPU)/PCL (outer/inner) grafts, while Xiao employed grafts with a heparinized PCL inner layer and a pristine PCL outer layer. While early patency was maintained in all animals, both studies reported progressive complications over time. Fang et al. observed occlusions after 3 months, and Xiao et al. reported aneurysmal changes and occlusion events between months 3 and 9, highlighting the challenge of sustaining long-term functionality in the rabbit model.^27,28^ The successful application of PCL-based vascular grafts in small animal models is not limited to these studies; findings from a broader range of research also support these outcomes.^20,29–35^ While these studies provide valuable insights, evaluating such grafts in large animal models remains a crucial step toward the development of clinically relevant vascular grafts that ensure long-term functionality and patency. However, there are some limitations in large animal model studies in the literature, such as ensuring long-term patency. For instance, Buscemi et al. reported thrombosis within 3 weeks in heparin-loaded PCL/PLA grafts implanted in porcines.^36^ Similarly, Mrówczyński et al. documented a 78% patency rate at 4 weeks with PCL grafts in a porcine model although follow-up was limited.^37^

In this study, bilayered electrospun vascular grafts with an inner layer composed of PCL/PLA and an outer layer of PLCL, as well as grafts composed entirely of PCL, are constructed by altering fiber orientations in different layers and tested both mechanically and biologically. After *in vitro* tests, grafts are implanted into the carotid arteries of porcines to evaluate long-term patency. Furthermore, the influence of the graft materials and systemic drug support on both the *in vitro* and *in vivo* performance is comprehensively investigated. By achieving partial graft patency up to 90 days in a porcine model, this study stands out as a rare and innovative example of long-term evaluation of electrospun vascular grafts in a large animal model.

## Materials and Methods

### Polymer Solution Preparation and Graft Fabrication

Biodegradable vascular grafts were fabricated using the electrospinning technique with PCL (*M_n_* 80,000), PLA (*M_n_* 230,000), and PLCL purchased from Sigma-Aldrich (USA). The bilayer tubular grafts consisted of a randomly distributed inner layer and a radially oriented outer layer. Inner layers were prepared from PCL or a PCL/PLA blend (80/20 wt %), while the outer layer was composed of either PCL or PLCL, depending on the sample type. Accordingly, the grafts were referred to as PCL_PCL and PCLPLA_PLCL, respectively. The selection of these polymers, blend ratios, and layer architectures was based on our previous studies demonstrating favorable mechanical properties and biocompatibility of monolayer and bilayer samples for vascular tissue engineering applications.^21,38,39^ A schematic representation illustrating the construction details of the monolayer and bilayer graft configurations produced at each stage of the study is provided in Figure 1.

**Figure 1:**
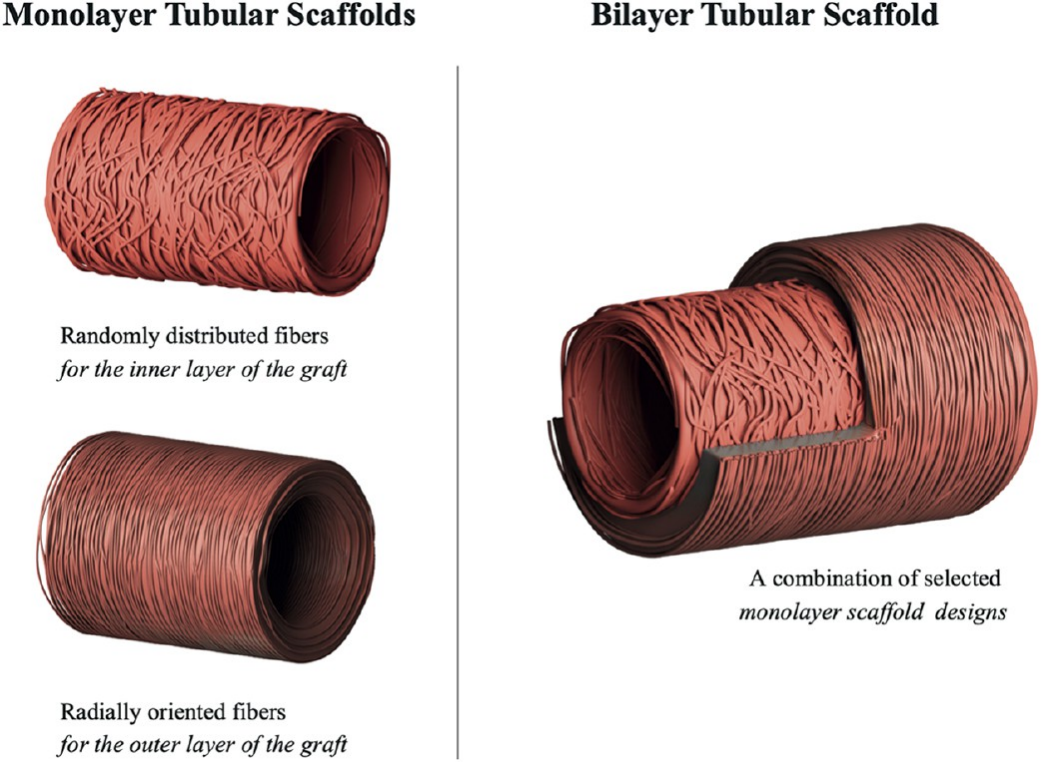
Architecture of fabricated electrospun monolayer and bilayer tubular scaffolds.

Solutions were prepared according to previous optimization^38,40,41^ in a chloroform/ethanol/acetic acid solvent system (8:1:1 wt %) at 8 wt % concentration for PCL/PLA blends and 10 wt % for PLCL. Electrospinning was performed using a modified closed-system electrospinning unit (Inovenso Nanospinner NE100+) equipped with a rotary mandrel. The inner layer was electrospun at 200 rpm for 20 min and the outer layer at 10,000 rpm for 55 min to obtain appropriate fiber orientation and wall thickness. The applied voltage was 12  ±  2 kV for PCL-based solutions and 10  ±  1 kV for PLCL, with a 20 cm needle-to-collector distance. All production parameters were optimized in our previous studies.^38–41^ The electrospinning environment was maintained at 21  ± 3 °C and 62  ±  8% relative humidity. The vascular graft samples were fabricated with inner diameters of 4, 5, and 6 mm and an approximate length of 7 cm. Produced grafts were dried at room temperature for 24 h before removal from the mandrel.

### Scanning Electron Microscopy (SEM)

Surface morphology of the electrospun vascular grafts and the suture regions after the suture retention test were evaluated using SEM (TESCAN VEGA3) at magnifications of ×200, ×300, ×500, and ×1000. Prior to imaging, all samples were sputter-coated with a gold–palladium (Au–Pd) alloy to enhance the conductivity. For the analysis of the vascular graft surfaces, ImageJ software was employed to determine fiber diameters; at least 50 fibers are measured per graft type. Wall thicknesses were also measured from the SEM images by using ImageJ software. Fiber orientation was analyzed using the Directionality plugin in Fiji (ImageJ). Orientation distributions were visualized through line graphs, where the *x*-axis represents the angular direction and the peak indicates the predominant fiber alignment. In samples with radial alignment, the peak is centered at 90°, whereas in samples with random orientation, it was centered at 0°. Additionally, postsuture retention test SEM imaging was performed to assess the structural integrity and surface morphology at the suture lines, allowing for visualization of potential fiber deformation, tearing, or morphological alterations induced by surgical manipulation.

### Sterilization Protocol

Prior to *in vitro* and *in vivo* studies, graft samples were sterilized to ensure biocompatibility. Grafts were washed in 70% ethanol, followed by three 15 min rinses in phosphate-buffered saline (PBS) (DPBS 1×, pH 7.4, Gibco). Subsequently, they were exposed to UV light in a laminar flow cabinet (Mars Safety Class 2, SCANLAF, Denmark) for 30 min per side to achieve thorough sterilization. Prior to cell seeding for cell viability and proliferation analyses using the MTS assay, the sterilized grafts were incubated overnight in a humidified incubator at 37 °C with 5% CO_2_.

### *In Vitro* Cell Culture and Cytocompatibility Assessment

For *in vitro* analysis, human aortic smooth muscle cells (SMCs) (T/G HA-VSMC, ATCC CRL-1999) and human umbilical vein endothelial cells (HUVEC, ATCC CRL-1730) were sourced from the American Type Culture Collection (ATCC, Manassas, VA, USA). Trypan Blue and Trypsin–EDTA (0.025% trypsin/1 mM EDTA) were obtained from Thermo Fisher Scientific. The following reagents were also employed for cell proliferation studies: phosphate-buffered saline (DPBS 1×, pH 7.4, Gibco, U.K.), penicillin/streptomycin (Gibco, Life Technologies, U.K.), fetal bovine serum (FBS, ATCC), F-12K medium (Kaighn’s Modification of Ham’s F-12 Medium, ATCC, Manassas, VA, USA), ascorbic acid (Sigma, USA), insulin (Gibco, U.K.), transferrin (Gibco, U.K.), sodium selenite (Gibco, U.K.), EBM-2 basal medium (CC-3156, Lonza), EGM-2 SingleQuots (CC-4176, Lonza), and the MTS assay reagent (CellTiter 96 AQueous One Solution Cell Proliferation Assay, Promega, USA).

HA-VSMCs were cultured in Ham’s F-12K medium (Kaighn’s Modification of Ham’s F-12 Medium, ATCC, Manassas, VA, USA) supplemented with 10% fetal bovine serum (FBS, ATCC) and additional components recommended by ATCC, while HUVECs were maintained in EBM-2 basal medium (CC-3156, Lonza) supplemented with EGM-2 SingleQuots (CC-4176, Lonza) following the manufacturer’s instructions. Both cell types were incubated under standardized conditions in a humidified incubator at 37 °C with 5% CO_2_. The culture medium was renewed every 3 days. Cells at passages 2 and 3 were utilized for all experiments to ensure cellular consistency.

For initial viability assessment and experimental standardization, cells were enzymatically dissociated using 0.025% trypsin/1 mM EDTA solution following a DPBS wash and incubated for 5 min at 37 °C to detach them from the culture surface. The resulting suspension (1 mL) is mixed 1:1 with 0.4% Trypan Blue solution (100 μL of cell suspension +100 μL of dye), and viable cells were counted manually using a Thoma hemocytometer (ISOLAB Laborgerate GmbH, Germany).

On the other hand, planar samples (1 × 1 cm^2^) were cut from tubular single-layer electrospun scaffolds for *in vitro* cytocompatibility and proliferation studies. Randomly oriented fibrous surfaces were used to represent the inner layer candidate and were seeded with HUVECs, while radially oriented fibrous surfaces representing the outer layer candidate were seeded with smooth muscle cells (SMCs). Bilayered tubular grafts were not used for the *in vitro* assays. Sterile samples were placed in 24-well plates prior to cell seeding. To ensure uniform distribution of the growth medium across the grafts and to monitor potential contamination, 500 μL of the appropriate growth medium was added to each well. The grafts were incubated in a cell-free environment for 24 h, after which the medium was aspirated. Subsequently, 1 × 10^5^ cells were seeded onto the sterile surfaces wetted with a growth medium. Prior to adding the growth medium, the grafts were incubated for 3 h at 37 °C in a 5% CO_2_ atmosphere to allow the cells to adhere to the grafts, preventing uncontrolled distribution within the wells. After incubation, the growth medium was carefully added, and the incubation process continued. The growth medium was refreshed every 3 days. On days 3, 7, and 14, the grafts were transferred to clean wells of a 24-well plate together with culture medium, and 100 μL of MTS reagent was added to each well to allow the formation of colored formazan by metabolically active cells, followed by incubation for 3 h. Subsequently, from the total volume of 600 μL, 200 μL was equally distributed into three wells of a 96-well plate, and the absorbance was measured at 490 nm using a microplate reader (Berthold Technologies, Germany). Cell viability was expressed relative to the control group (cells cultured in wells), which was considered 100%. All experiments were conducted in triplicate.

### Mechanical Characterization

#### Tensile Strength

Tensile testing was performed on grafts cut into rectangular strips (10 × 15 mm^2^) in both axial and circumferential directions as well as in tubular form (1.5 cm length). Tests were conducted by using a universal testing machine (ZwickRoell Z005, Germany) with a 200 N load cell at a strain rate of 10 mm/min.

#### Suture Retention Strength

Suture retention strength was assessed on 1 cm-long tubular grafts sutured end-to-end using a 7/0 polypropylene monofilament via a continuous suture technique. Tests were conducted using a universal testing machine (ZwickRoell Z005, Germany) with a 200 N load cell at a strain rate of 10 mm/min.

#### Burst Pressure and Compliance

To evaluate the mechanical durability and physiological compatibility of the vascular grafts, burst pressure and compliance tests were also conducted using a custom-built test apparatus designed in accordance with ISO 7198:2016 and introduced in our previous study.^39^ For burst pressure measurements, tubular samples (4 cm length) were sealed at both ends and internally pressurized with air until rupture. The peak pressure recorded at the point of failure was noted as the burst pressure.

Compliance testing was performed using the same setup under pulsatile pressure conditions, simulating a physiological range of 80–120 mmHg (normal blood pressure). A syringe pump generates controlled intraluminal pressure variations, and the graft diameter changes were tracked via a camera system. Compliance was calculated based on the relative change in diameter per 100 mmHg pressure difference using the following equation^39^

1 $$\%\;\mathrm{compliance}=\frac{\frac{R_{p_{2}}-R_{p_{1}}}{R_{p_{1}}}}{p_{2}-p_{1}}\times 10^{4}$$

where *R*_*p*1_ and *R*_*p*2_ represent the pressurized radii at diastolic (*p*_1_) and systolic (*p*_2_) pressures, respectively (in mm and mmHg). All compliance tests were conducted in triplicate for each graft sample to ensure statistical reliability.

### Surgical Procedure

Within the scope of the *in vivo* studies, male white Yorkshire pigs (*n* = 4), each aged 5–6 months and initially weighing 22–24 kg (ABDEHAM, Bursa, Turkey), were used. The *in vivo* studies were approved by the Local Ethics Committee for Animal Experiments of Bursa Uluda University (Approval No: 2021-10/06) and the Local Ethics Committee for Animal Experiments of the Istanbul Mehmet Akif Ersoy Experimental Research, Development, and Training Center (Protocol No: 2024/18; Date: 27.09.2024). All procedures were conducted in accordance with national regulations and ethical standards for the care and use of laboratory animals.

#### Preoperative Procedure

Animals were fasted for 12 h prior to surgery. Intramuscular premedication consisted of atropine (0.04 mg/kg; Atropin Sülfat, Galen laç, stanbul, Türkiye), followed by xylazine (3 mg/kg; Rompun, Bayer, stanbul, Türkiye) and ketamine (20 mg/kg; Alfamine, Alfasan, Netherlands), administered at 10 min intervals. Anesthesia was induced via intravenous administration of propofol (2 mg/kg; Propofol-Lipuro, B. Braun Medical Inc., Melsungen, Germany) through a 22G auricular vein catheter, followed by endotracheal intubation. Anesthesia was maintained with 2–4% isoflurane (Isoflurane, Adeka, Samsun, and Türkiye) in oxygen. Intraoperative monitoring included electrocardiography (ECG), cardiography, and pulse oximetry. Prophylactic antibiotics (amoxicillin/clavulanic acid, 20 mg/kg IM; Teknovet, stanbul, Türkiye) and analgesics (meloxicam, 0.5 mg/kg IV; Bavet, stanbul, Türkiye) were administered preoperatively.

#### Intraoperative Procedure

In the porcine models, 1.5 cm segments of the right common carotid artery were resected following proximal and distal vascular clamping. Electrospun grafts (PCL_PCL and PCLPLA_PLCL) with a diameter of 4 mm and a length of 2 cm were implanted using an interposition grafting technique in the first two subjects, whereas a bypass procedure was performed in the subsequent two subjects. For the bypass, the intervening arterial segment was permanently ligated using silk sutures to induce occlusion with vertical arteriotomies created proximal and distal to the ligated segment. The ends of the vascular grafts were trimmed into a fish-mouth configuration and anastomosed to the native artery in a side-to-end fashion, employing continuous 7/0 polypropylene sutures.

#### Postoperative Procedure

Following implantation, all animals were transferred to a postoperative care unit and monitored daily until suture removal. Postoperative management included intramuscular amoxicillin–clavulanic acid (20 mg/kg), administered every other day for a total of three doses, and intravenous meloxicam (0.5 mg/kg) for analgesia.

To minimize the thrombosis risk, distinct antithrombotic regimens were applied to the subjects. In Subject II, dual antiplatelet therapy comprising orally administered aspirin and clopidogrel was initiated in the early postoperative period. Subjects III and IV received a daily intradermal injection of low-molecular-weight heparin (enoxaparin, 4000 anti-Xa IU) starting from the day of surgery and continuing until euthanasia. Subject I did not receive any form of antithrombotic therapy. This differentiated pharmacological approach allows for the comparative evaluation of their respective effects on graft patency and thrombotic outcomes. Table 1 presents implanted samples, implantation methods, and perioperative pharmacological interventions.

**Table 1:** Summary of Graft Characteristics, Implantation Procedures, and Systemic Medication Regimens

animals	implanted sample	implantation method	systemic anticoagulant/antiplatelet drug
Subject I	PCLPLA_PLCL	replacement	
Subject II	PCLPLA_PLCL	replacement	aspirin + clopidogrel
Subject III	PCL_PCL	by-pass	clexane (enoxaparin)
Subject IV	PCL_PCL	by-pass	clexane (enoxaparin)

### Doppler Ultrasound Assessment

Patency, flow dynamics, and structural integrity of the implanted vascular grafts were assessed using high-resolution Doppler ultrasonography through a comparative evaluation of the treated carotid artery and the contralateral carotid artery under general anesthesia. Following bilateral cervical region shaving, longitudinal and transverse imaging of the cervical segment of the common carotid artery along the *sulcus jugularis* was performed through transcutaneous contact, using surgical spirit and ultrasound gel. A 12S phased-array pediatric cardiac and coronary probe (4.0–12.0 MHz) was used in conjunction with a GE Vivid S60S echocardiography system (General Electric, Norway). The obtained images were recorded and analyzed by using the EchoPAC PC clinical workstation software. Measurements included arterial diameters from longitudinal views and Doppler velocity profiles, namely, peak systolic velocity (PSV) and end diastolic velocity (EDV), expressed in cm/s. Postsurgical imaging was performed on days 3 and 10 for Subjects I and II and on days 3, 10, 40, 50, and 90 for Subjects III and IV. Moreover, graft patency was defined as the midgraft lumen diameter measured by Doppler ultrasound. All vascular grafts had an initial inner diameter of 4 mm. Patency (%) was calculated by comparing the midgraft diameter at each follow-up to the initial diameter.

### Sacrifice and Explantation

All subjects underwent euthanasia and graft explantation procedures in accordance with ethical guidelines, particularly in cases where the graft patency rate falls below 20% or when concerns regarding the animal’s health status are identified. Animals were first sedated using intramuscular administration of ketamine and xylazine, followed by intravenous injection of 10 mL of 2% propofol via the lateral auricular vein. Once deep anesthesia was confirmed, euthanasia was completed by intracardiac injection of potassium chloride to ensure humane termination.

Following euthanasia, the implanted vascular grafts were carefully dissected from the surrounding tissue. In select cases, the contralateral carotid artery was also harvested to serve as a comparative control for mechanical and histological analyses. Mechanical analysis of the native carotid arteries was performed within 24 h following explantation, without the application of any fixation procedure. On the other hand, both grafts and the carotid arteries were rinsed and immersed in 10% neutral-buffered formalin to remove adipose tissue and to prepare the samples for histological evaluation.

### Postmechanical Assessment

The tensile testing was also employed on the contralateral carotid arteries explanted from subjects in their native tubular form (1.5 cm length) along the axial direction for further comparison. Similar to graft testing, carotid artery testing was also conducted by using a universal testing machine (ZwickRoell Z005, Germany) with a 200 N load cell at a strain rate of 10 mm/min.

### Histological Analysis

Hematoxylin and eosin (H&E) staining was performed to examine overall tissue morphology, including cellular infiltration, neointimal formation, fibrosis, necrosis, and inflammatory processes. For control, the contralateral carotid artery was also examined.

The excised vessels and vascular grafts were fixed in 10% neutral-buffered formalin at 4 °C, rinsed in phosphate-buffered saline (PBS, pH 7.4), dehydrated through a graded ethanol series, cleared in xylene, and embedded in paraffin. Serial sections of 3 μm thickness were obtained by using a rotary microtome. Sections were deparaffinized, rehydrated through descending ethanol concentrations, stained with Mayer’s hematoxylin, blued in Scott’s tap water substitute, and counterstained with eosin. After dehydration and clearing, the sections were mounted with an Entellan microscope (Merck) and examined under a light microscope (ECLIPSE Ci Series, Nikon, NY, USA). Histological evaluation for cellular infiltration, neointimal formation, fibrosis, necrosis, and inflammatory processes was performed by experienced pathologists in a blinded manner.

### Statistical Analysis

MTS assay and mechanical analysis results were statistically analyzed using Student’s *t*-test in Minitab 16 software. Statistical analyses were performed at a 95% confidence level, and a *p* < 0.05 is considered statistically significant.

## Results and Discussion

### SEM Analysis

SEM images of the layer surfaces and graft cross sections, the fiber directionality graphs of the inner and outer surfaces, and the fiber diameter and wall thickness values are presented in Table 2. As observed, the fabricated grafts exhibited homogeneous and continuous fiber morphology in both inner and outer layers. A comparison of the fiber diameters in the inner layers revealed that the PCLPLA blend exhibited a greater average fiber diameter than the PCL layer (1.57 vs 1.17 μm, respectively (*p*-value: 0.031 < 0.05)). This increase can be primarily attributed to the incorporation of PLA, which possesses a higher molecular weight than PCL. The elevated molecular weight enhances the viscoelasticity of the spinning solution, thereby leading to thicker fibers during electrospinning.^21,42,43^ In contrast, the outer PCL layer, fabricated using a higher collector linear speed, displayed a smaller fiber diameter (1.04 μm) compared to its inner counterpart (1.17 μm) although the difference is not statistically significant (*p*-value: 0.453 > 0.05). The increased rotational speed of the collector induces additional stretching of the deposited fibers, which, in turn, results in thinner fiber formation.^44,45^ On the other hand, SEM images of the outer layers demonstrated a pronounced radial alignment of the fibers, which became more evident with increasing collector speed, as further supported by the fiber orientation analysis. The elevated linear velocity of the rotating mandrel at higher speeds facilitates the alignment of fibers along the circumferential direction.^46^

**Table 2:**
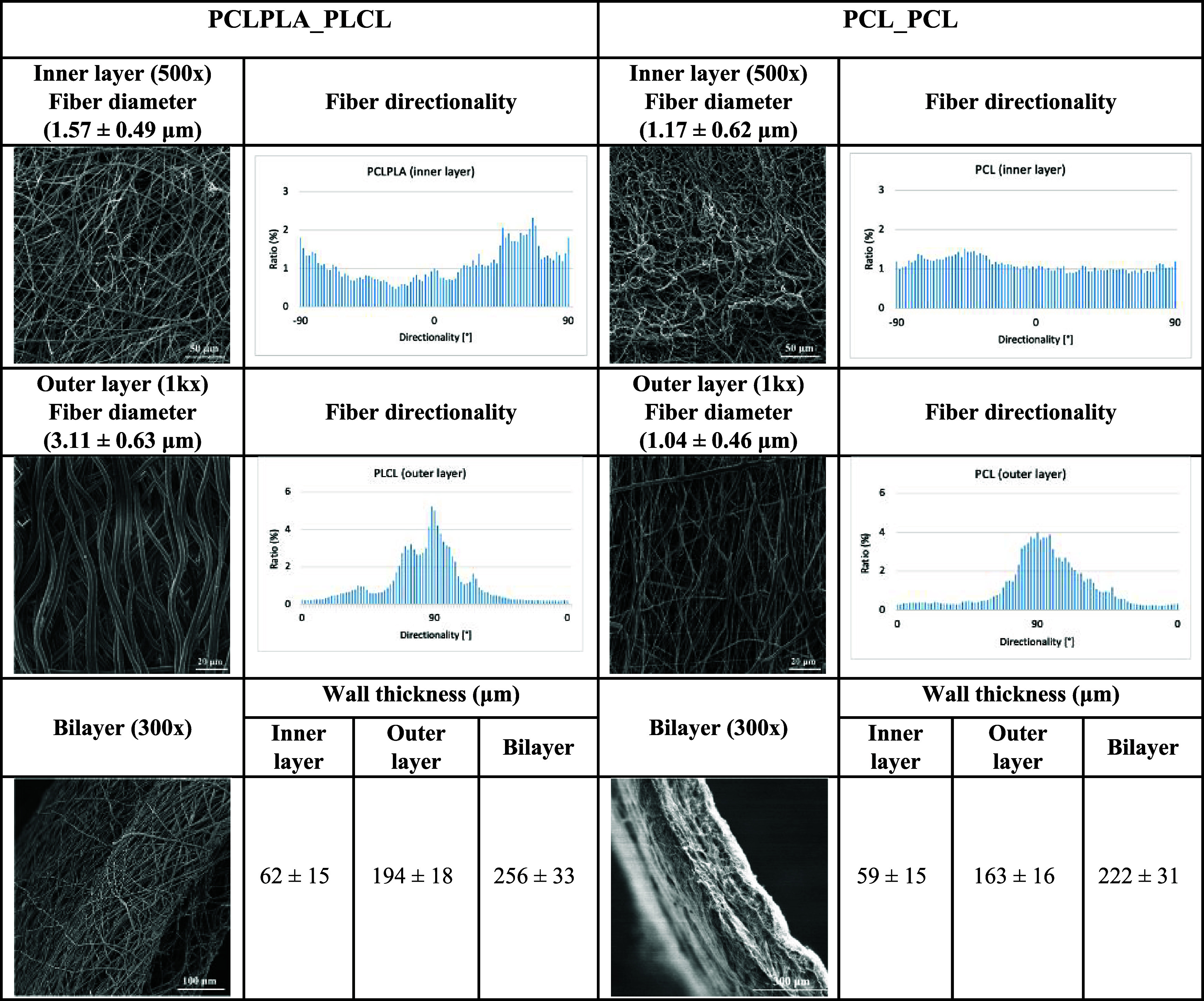
SEM Micrographs, Fiber Diameter, and Wall Thickness Measurements of the Fabricated Vascular Grafts

Although the layers were successfully fabricated, the PCLPLA_PLCL graft showed microscale delamination at the interface, most likely due to material and structural incompatibilities between the dissimilar layers, resulting from insufficient intermolecular adhesion.^47^ In contrast, the PCL_PCL graft demonstrated seamless integration between layers, which can be attributed to the use of the same polymer in both regions, ensuring better interfacial compatibility.^31^ Furthermore, the measured wall thicknesses of all grafts fell within the 200–600 μm range for *in vivo* evaluation.^48^ Graft wall thickness was predefined during fabrication based on ranges commonly reported in the literature for porcine carotid artery implantation (250–500 μm).^49,50^ It is also observed that the inner layers of the fabricated scaffolds exhibited a lower wall thickness compared to the outer layers, which aligns with the structural design strategy of this study. The wall thicknesses of the inner layers measured 62 μm for PCLPLA_PLCL and 59 μm for PCL_PCL, while the outer layers displayed thicknesses of 194 and 163 μm, respectively. In the literature, the *tunica intima* of the human carotid artery was reported to range from 68 to 95 μm,^51^ whereas the *tunica media* had a greater thickness, typically between 125 and 350 μm.^52^ These findings indicated that the obtained thickness values closely approximated the native arterial wall and were suitable for mimicking the structure of the carotid artery.

### MTS Cell Proliferation Assay

The cell proliferation rates of the grafts cultured with HUVECs and HA-VSMCs on days 3, 7, and 14 are shown in Figure 2. Surfaces cultured with HUVEC represent the inner layer of the bilayer vascular graft, while those cultured with HA-VSMC represent the outer layer. Among the grafts cultured with HUVECs, both PCL and PCLPLA showed a steady increase in cell proliferation from day 3 to day 14. On the final day, their viability levels reach 121.16 and 126.42%, respectively, with both values significantly higher than those of the control group (*p* < 0.05). Notably, HUVEC proliferation on PCLPLA remained consistently higher than that observed on neat PCL throughout the culture period, suggesting a positive contribution of PLA incorporation to endothelial cell viability. This finding is in line with our previous results, where the addition of 20% PLA to PCL increased HUVEC viability by approximately 30%.^21^ Similarly, Sadiasa et al. reported that PCL/PLLA blend surfaces promoted cell viability compared to neat PCL substrates.^53^

**Figure 2:**
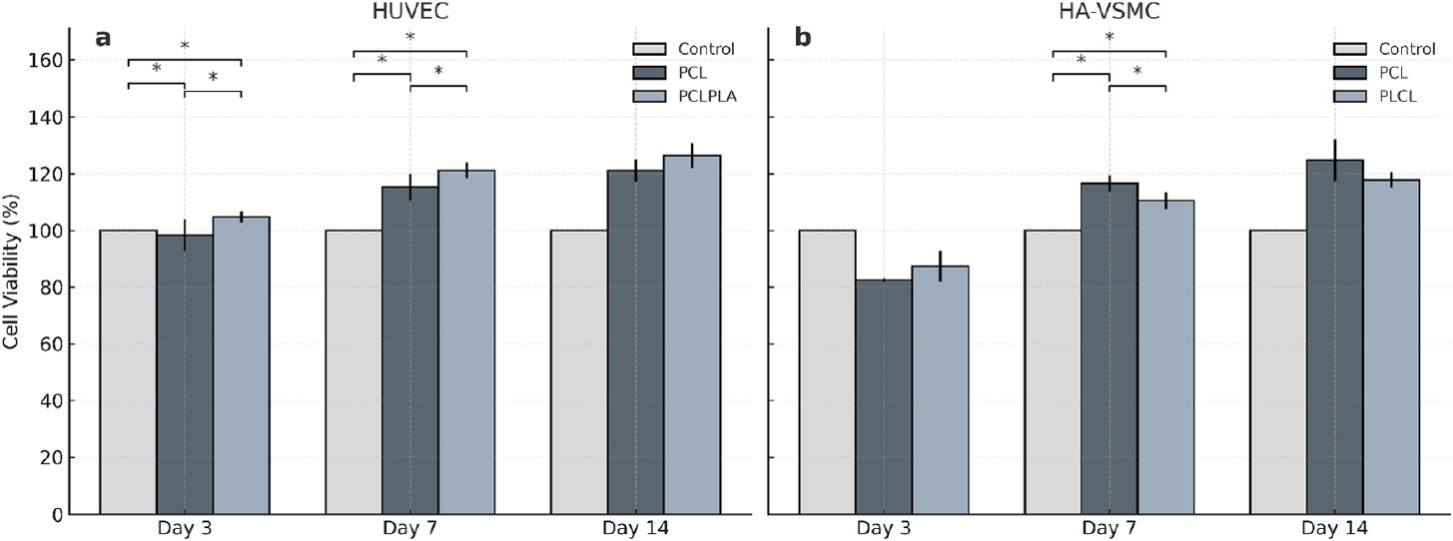
MTS analysis of the surfaces cultured with HUVECs (a) and HA-VSMCs (b) on the 3rd, 7th, and 14th days. *Indicates the statistically significant difference at a 95% confidence interval.

On HA-VSMC-cultured grafts, initial viability rates were lower than 100%, reflecting a decrease relative to the control. However, a significant increase was observed as the incubation period advanced (*p* < 0.05). These results highlight the role of porous and fibrous architectures in creating a three-dimensional microenvironment that facilitates HA-VSMC proliferation. On the other hand, it was observed that PCL provides a more suitable environment for smooth muscle cell existence on the seventh and 14th days compared to PLCL. Overall, the grafts cultured with both HUVECs and HA-VSMCs are all found to support cell viability and metabolic activity.

### Mechanical Assessments

#### Tensile Stress and Strain

The tensile stress and strain curves of the bilayer vascular grafts are presented in Figure 3, highlighting their mechanical suitability for further *in vivo* investigation. The quantitative tensile strength and elongation values obtained from the tests are additionally summarized in Table 3. The bilayer samples exhibited superior tensile strength in the radial direction (7.03 and 10.21 MPa for PCL_PCL and PCLPLA_PLCL, respectively) compared to the axial direction, both in planar (2.19 (*p*-value: 0.013 < 0.05) and 2.72 MPa (*p*-value: 0.001 < 0.05), respectively) and tubular (2.31 (*p*-value: 0.005 < 0.05) and 3.05 MPa (*p*-value: 0.005 < 0.05), respectively) forms. This marked difference suggests that the radially aligned fibers in the outer layer effectively mimic the mechanical reinforcement function of *tunica media* in native vessels. The results underscore the significance of fiber orientation, with tensile strength and elongation being notably higher along the direction of fiber alignment. This enhancement is likely due to the collective resistance of aligned fibers to applied loads, thereby improving mechanical performance in the radial direction.^23,54–56^ Moreover, the PCLPLA_PLCL graft exhibited higher stress and strain values in all tested directions, which can be attributed to the incorporation of PLA in the graft structure, enhancing the overall mechanical performance.^57^ Notably, the stress–strain curves of PCLPLA_PLCL, containing a PCLPLA inner layer, displayed two distinct peaks in all directions. The first peak corresponds to the failure of the inner layer, while the second peak indicates the rupture of the outer layer. In contrast, such a dual-peak pattern was not observed in the PCL_PCL graft. Instead, PCL_PCL showed more uniform curve profiles and no significant signs of delamination during tensile testing, indicating better structural compatibility, as also supported by SEM analysis. The presence of dual peaks in the PCLPLA_PLCL curves thus reflected a mechanical and structural mismatch between the layers, leading to delamination. Overall, the stress–strain behavior of bilayer grafts represented a composite response arising from the individual mechanical contributions of each layer.^23,39^

**Figure 3:**
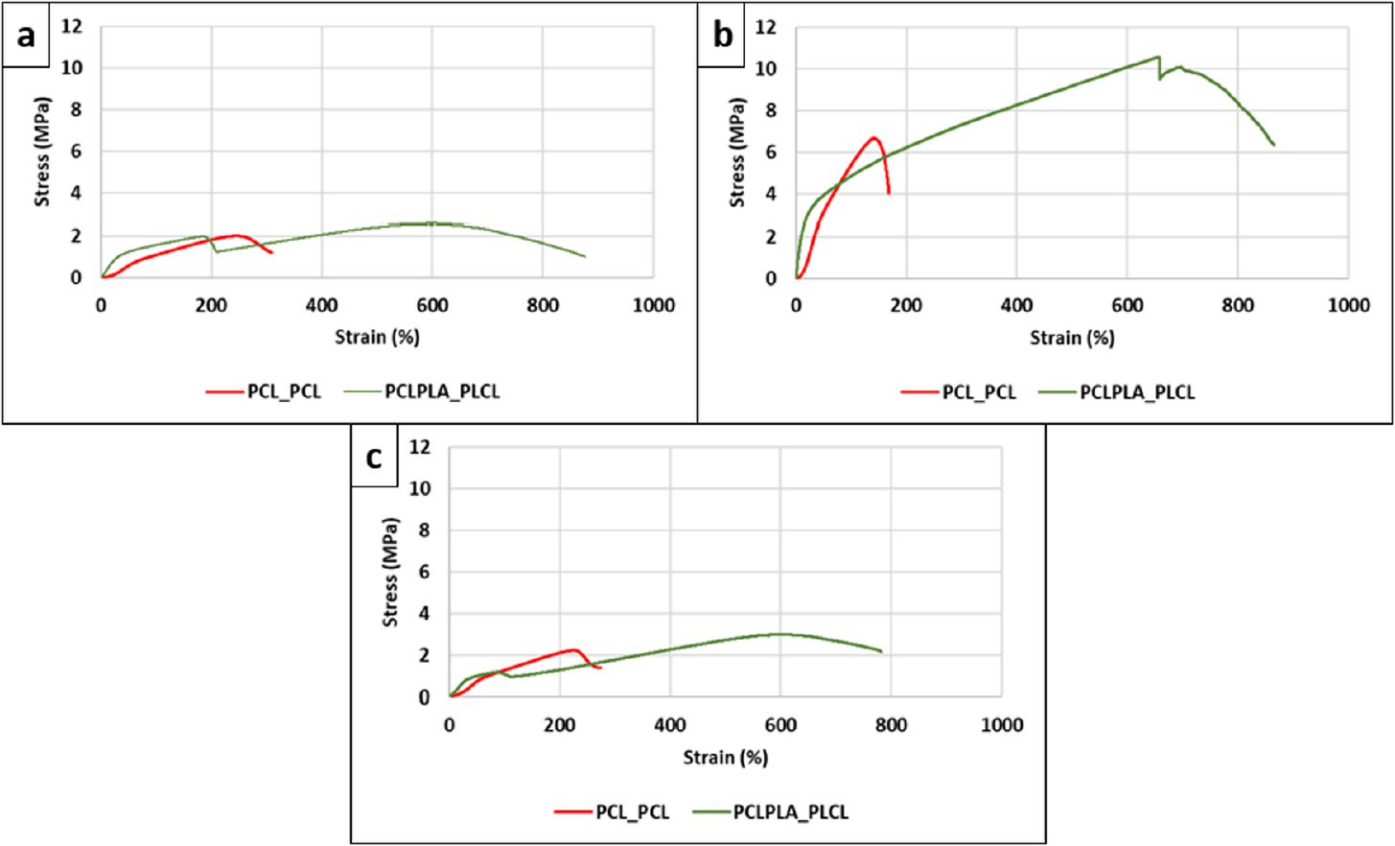
Tensile stress–strain curves of PCL_PCL and PCLPLA_PLCL samples: (a) axial direction in planar form, (b) radial direction in planar form, and (c) axial direction in tubular form.

**Table 3:** Tensile Strength and Strain Values of Bilayer Vascular Grafts

	PCLPLA_PLCL			PCL_PCL		
	axial (planar)	circumferential (planar)	axial (tubular)	axial (planar)	circumferential (planar)	axial (tubular)
tensile strength (MPa)	2.72 ± 0.61	10.21 ± 0.6	3.05 ± 0.36	2.19 ± 0.41	7.03 ± 0.89	2.31 ± 0.63
elongation (%)	457.81 ± 143.57	777.39 ± 125.60	588.56 ± 154.14	249.54 ± 29.44	122.53 ± 17.37	201.08 ± 21.65

### Suture Retention Strength

Table 4 presents the results of the suture retention test along with SEM images taken from the suture regions of the bilayer grafts after testing. Examination of the stitched areas revealed that although the samples exhibited localized damage, complete tearing did not occur. Instead, the grafts were observed to loosen only around the suture sites without undergoing full structural failure.

**Table 4:**
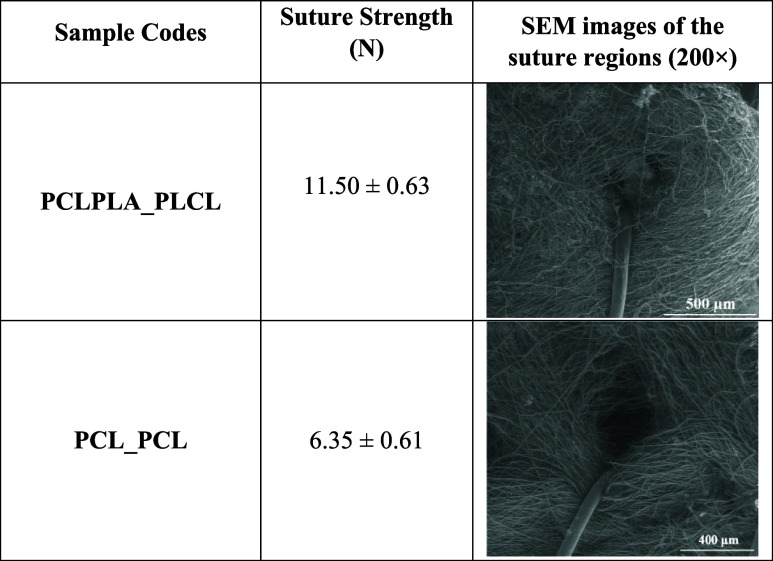
Suture Retention Strength Values and SEM Images of the Suture Regions of the Samples

Upon evaluation of the suture retention test results, it was determined that the PCLPLA_PLCL and PCL_PCL samples exhibited suture retention strength values of 11.5 and 6.35 N, respectively. These values indicated a superior mechanical performance when compared to the human saphenous vein, commonly regarded as the clinical gold standard, which typically demonstrates suture retention strengths in the range of 2–3 N.^58^

### Burst Pressure and Compliance

The burst pressure and compliance values of the grafts produced for implantation are listed in Table 5. Considering that the human saphenous vein exhibits a burst strength of approximately 1500 mmHg and a commercial ePTFE vascular graft typically withstands around 1300 mmHg,^59^ the bilayer grafts developed within the scope of this study demonstrated highly satisfactory mechanical performance (1641–2021 mmHg, Table 5). Given that burst resistance is primarily governed by pressure exerted in the radial direction, it can be inferred that the outer layer predominantly contributes to the structural reinforcement. This observation is consistent with numerous reports in the literature highlighting that multilayer graft architectures offer superior mechanical properties compared to monolayer designs.^23,38,60,61^

**Table 5:** Burst Pressure and Compliance Values of Bilayer Tubular Grafts

	burst strength (mmHg)	compliance (%/100 mmHg)
PCLPLA_PLCL	2021.00 ± 198.50	0.81 ± 0.36
PCL_PCL	1641.50 ± 79.50	2.67 ± 0.49

The PCLPLA_PLCL graft exhibited higher burst strength than the PCL_PCL graft (*p*-value: 0.012). This improvement was largely attributed to the incorporation of PLA, a polymer known for its outstanding mechanical strength and intrinsic stiffness.^39^

When the compliance values of the fabricated vascular grafts were compared, the PCL_PCL graft demonstrated a higher compliance (2.67%/100 mmHg) than the PCLPLA_PLCL graft (0.81%/100 mmHg). This reduction in compliance was attributed to the incorporation of PLA into the inner layer, which increased the overall stiffness of the construct due to the inherently brittle and rigid nature of PLA.^39^ For reference, the compliance values of commercially available synthetic grafts such as Dacron and ePTFE typically range between 0.26 and 1.9%/100 mmHg, which is comparable to or lower than that of the human saphenous vein (∼1.5%/100 mmHg).^62–64^ In contrast, arteries such as the human coronary artery exhibit considerably higher compliance, with values reaching approximately 3.8%/100 mmHg.^65^ Accordingly, the compliance of PCL_PCL grafts not only exceeded that of the saphenous vein but also approached the compliance of the coronary artery, suggesting favorable biomechanical compatibility with native vasculature. On the other hand, the compliance of PCLPLA_PLCL grafts remained within or just above the upper range of commercial grafts, yet fell below that of native arteries. This mechanical mismatch might have contributed to the increased risk of graft failure due to compliance mismatch at the anastomosis sites.^66^

Therefore, when the burst pressure and compliance results were evaluated together, it became apparent that while PLA significantly enhanced mechanical strength, its relatively stiff characteristics compromised the flexibility required for optimal vascular graft performance.

### Surgical Procedure

The surgical procedure was successfully performed by implanting the bilayer graft designs (PCLPLA+PLCL and PCL+PCL) into the carotid arteries of porcines by using either replacement or bypass techniques. A preoperative image of the target carotid artery and an intraoperative view of the implanted graft are shown in Figure 4.

**Figure 4:**
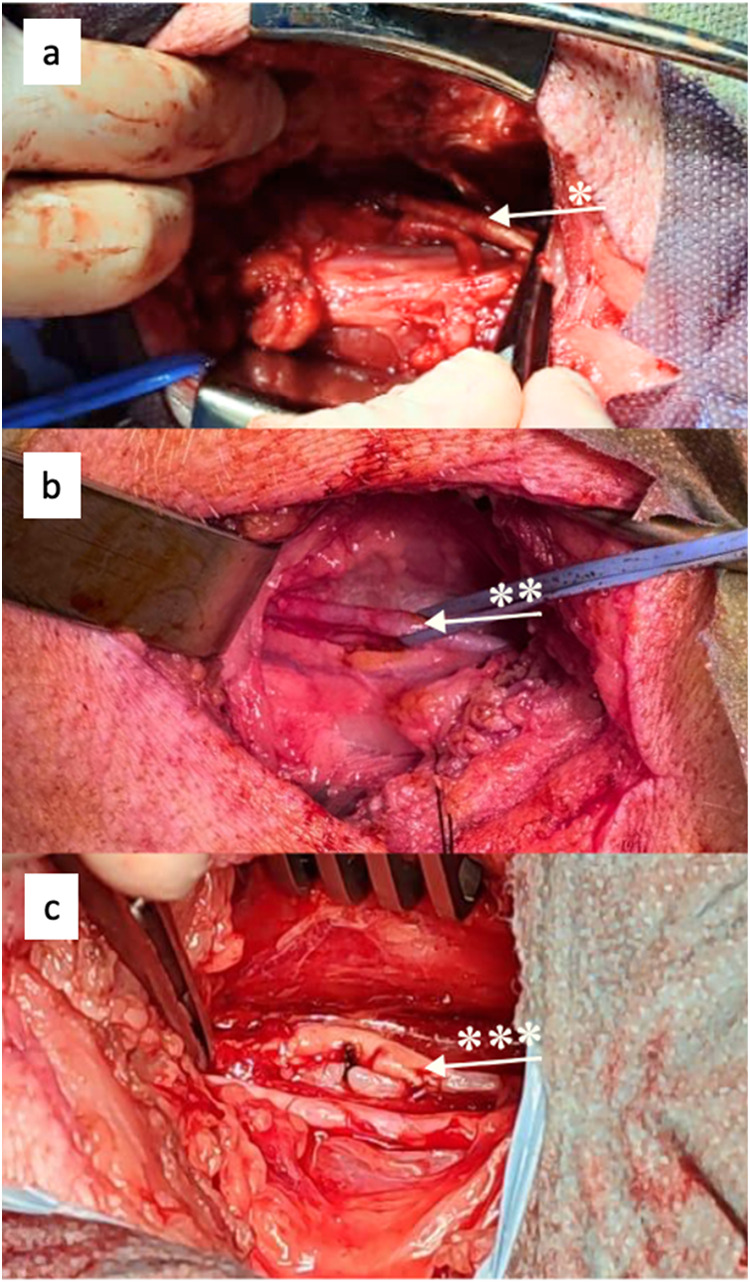
(a) Intraoperative view of the implanted bilayer vascular graft via the replacement method. (b) Preoperative image of the carotid artery targeted for the bypass. (c) Intraoperative view of the implanted bilayer vascular graft via the bypass method. The bypassed carotid artery is marked with ** and the implanted graft with * and ***.

### Doppler Ultrasound Assessment

The patency rates of vascular grafts and Doppler ultrasound images of both control groups and implanted grafts are listed in Figure 5. According to the results, Subject I and Subject II showed similar perivascular complications following graft implantation. In Subject I, Doppler evaluation on postoperative day 3 revealed marked narrowing of the carotid artery and reduced flow velocities (Table 6). By day 10, almost complete thrombotic occlusion had occurred. A firm area of tissue swelling was noted upon clinical examination around the surgical site, and ultrasonography demonstrated a large anechoic fluid collection, from which serosanguineous fluid was aspirated. In Subject II, despite the administration of aspirin and clopidogrel, progressive narrowing at the graft site was observed on postoperative days 3 and 10. While approximately 38% patency was maintained on day 3, it had declined to below 20% by day 10, similar to that observed in Subject I. Ultrasound imaging identified hyperechoic longitudinal structures consistent with organized thrombus. Additionally, extensive edema extending into the submandibular region and hematoma-like formations (Figure 6) suggested a possible graft-related leakage. In both cases, edema, fluid accumulation, and inflammatory responses likely compromised graft function and contributed to early occlusion. Notably, in Subject II, a transient narrowing (∼4%) was also detected in the contralateral, nonoperated carotid artery on day 3, attributed to suboptimal hydration. However, the vessel diameter returned to baseline by day 10. In Subject II, an increase in blood flow velocity was observed, along with vascular constriction.

**Figure 5:**
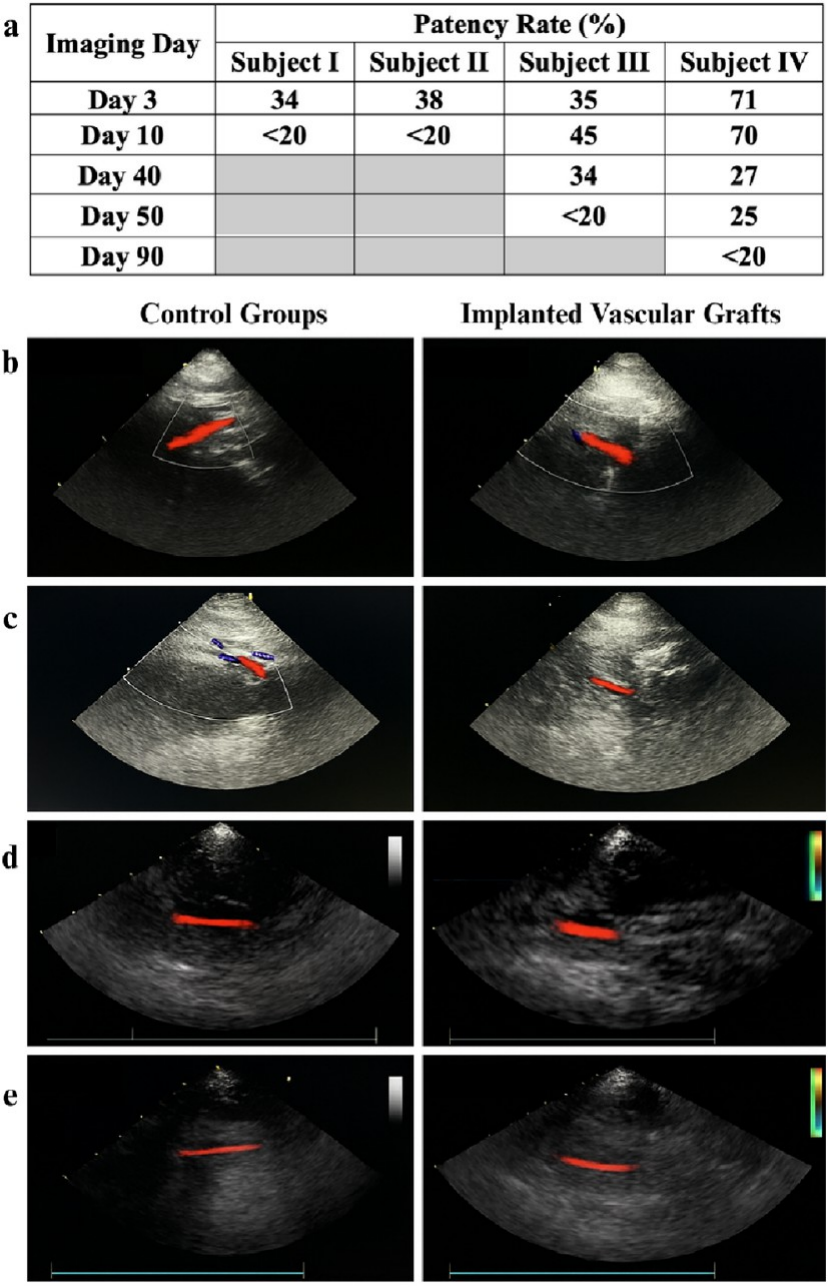
(a) Patency rates and Doppler ultrasound Color Flow Motion (CFM) images of control groups and vascular grafts on day 3: (b) Subject I, (c) Subject II, (d) Subject III, and (e) Subject IV. Color overlays in images (d, e) are added using AI-based visualization to enhance blood flow clarity. Original grayscale Doppler images are retained.

**Figure 6:**
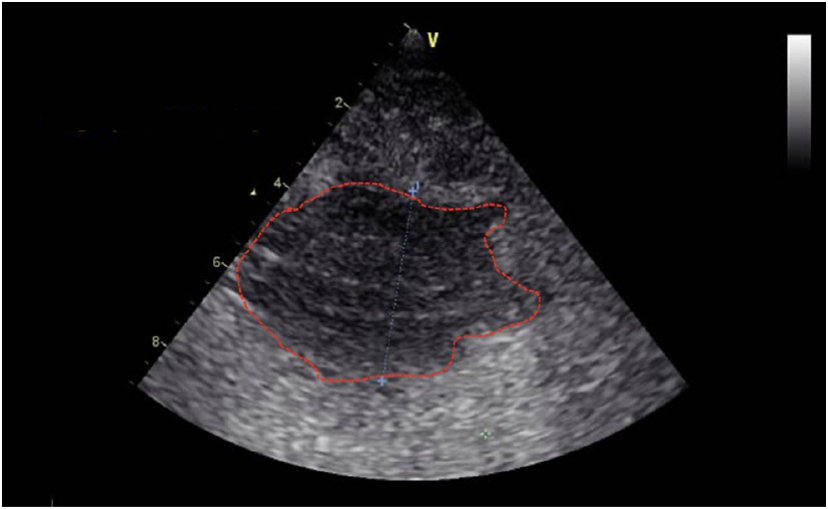
Hematoma-like hypoechoic area formed around the operation area.

**Table 6:** End Diastolic and Peak Systolic Velocities of Nonoperated Vessels and Operated Vascular Grafts of Subject I and Subject II

	end diastolic velocity (cm/s)				peak systolic velocity (cm/s)			
	Subject I		Subject II		Subject I		Subject II	
imaging day	contralateral carotid	implanted graft	contralateral carotid	implanted graft	contralateral carotid	implanted graft	contralateral carotid	implanted graft
day 3	23.02	39.51	108.78	74.94	51.12	43.72	135.15	88.32

During the implantation of the graft (PCLPLA_PLCL) in Subject I, partial delamination was intraoperatively observed at the interface between the inner and outer layers. This structural instability is believed to have significantly contributed to the early occlusion observed on postoperative day 3. Although no visible delamination was detected during the implantation of the second graft of the same type (PCLPLA_PLCL) in Subject II, whether interfacial separation may have developed during the follow-up period. Notably, the antiplatelet medication (aspirin and clopidogrel) administered to Subject II did not appear to have a beneficial effect, as graft patency was maintained only until day 10, similar to that of Subject I. Similar concerns have been reported in the literature. Atari et al. demonstrated that interlayer delamination in trilayer vascular grafts resulted in *in vivo* failure, underscoring the importance of interfacial stability.^67^ In contrast, in the PCL_PCL grafts implanted in Subjects III and IV, no delamination or interfacial instability was observed either intraoperatively or postoperatively. This finding is also consistent with monopolymer usage and is supported by the SEM cross-sectional images (Table 2).

Postoperative follow-up data from Subject III and Subject IV initially revealed similar pathophysiological responses to graft implantation but diverged in clinical progression over the mid- to long-term. In both cases, early postoperative evaluations indicated perivascular edema, inflammation, and fluid accumulation; however, the severity and implications of these findings differed substantially. For instance, on day 3, Subject III exhibited pronounced edema, traumatized tissue, and limited graft patency measured at 35%. In contrast, Subject IV demonstrated a more favorable profile with 71% patency and a continuous laminar Doppler signal. In subsequent assessments, while edema regressed and fluid collections showed signs of organization in both models, graft patency in Subject III improved only modestly to 45%. In comparison, Subject IV maintained a stable 70%. During this period, blood flow in Subject III remained weak and intermittent, whereas Subject IV sustained a more regular and continuous flow pattern (Table 7). On the other hand, by the midterm follow-up, particularly from day 40 onward, a marked decline in patency was observed in Subject III, dropping from 34% to below 20% at day 50, accompanied by diminished Doppler signals and loss of normal waveforms. However, Subject IV showed a more gradual reduction (from 27 to 25%) while maintaining flow, although perivascular abscess formation raised concerns about localized infection. Imaging quality during this phase posed a challenge, limiting interpretability. Subject IV successfully maintained partial patency at day 90; however, the process was terminated because this value remained below 20%.

**Table 7:** End Diastolic and Peak Systolic Velocities of Nonoperated Vessels and Operated Vascular Grafts of Subject III and Subject IV

	end diastolic velocity (cm/s)				peak systolic velocity (cm/s)			
	Subject III		Subject IV		Subject III		Subject IV	
imaging day	contralateral carotid	implanted graft	contralateral carotid	implanted graft	contralateral carotid	implanted graft	contralateral carotid	implanted graft
day 3	62.05	73.37	58.98	57.25	108.10	146.60	126.89	106.84
day 10	65.71	65.71	59.78	68.30	108.83	132.98	152.96	156.90
day 40	43.55	44.55	50.12	16.36	74.61	90.15	89.38	44.83
day 50	33.54	33.00	36.32	13.25	68.40	101.48	68.24	64.50
day 90			38.95	30.84			121.77	75.59

In the literature, large animal models are generally followed up for shorter periods, and occlusion is frequently encountered. For example, in a study by Zhai et al., vascular grafts consisting of a P­(LLA-CL) shell and a heparin-loaded core were fabricated using coaxial electrospinning. After implantation into canine femoral arteries, both the nonheparin-loaded and endothelialized P­(LLA-CL) grafts were completely occluded by day 30, whereas the heparin-loaded grafts demonstrated patency rates of 100% at day 7, 50% at day 14, and 25% at day 30.^68^ In another study, Fang et al. reported that electrospun PCL grafts implanted into sheep carotid arteries without anticoagulant treatment lost patency at day 7. In contrast, enoxaparin-treated grafts remained patent for up to 24 days.^69^ Porcine models closely resemble human vascular anatomy; however, their high thrombogenicity and strong immune responses pose challenges for long-term *in vivo* testing.^37^ In this context, the prolonged graft patency observed in Subject IV stands out as a remarkable achievement in large animal implantation studies. These findings underscore the importance of both structural integrity and adequate pharmacological support in the design of vascular grafts. On the other hand, despite using the exact same graft and receiving identical medical care, the patency rates of Subject III and Subject IV differed. This indicates that even with identical graft designs, individual anatomical and physiological variations can significantly influence *in vivo* performance.

Although variability exists in the *in vivo* experimental design with respect to material type, surgical approach, drug regimen, and follow-up duration, internal consistency was preserved within each surgical scenario. However, this design does not allow for strict isolation of individual parameters, and the effective sample size per condition remains limited. Therefore, the *in vivo* findings should be interpreted as exploratory observations reflecting graft behavior across distinct clinically relevant scenarios rather than definitive evidence of isolated material or pharmacological effects.

### Explantation

Table 8 presents the explantation time points determined based on Doppler ultrasonography findings indicating luminal patency below 20%, along with the explantation status of the graft and the contralateral carotid artery, as well as their subsequent use in mechanical and histological analyses. Although both the graft and the contralateral carotid artery were intended to be retrieved from each subject for further evaluation, this was not feasible in all cases due to limitations such as tissue integration and dissection-related challenges.

**Table 8:** Explantation Time, Graft and Contralateral Carotid Explantations, and Postmechanical and Histological Analysis Availability

				postmechanical analysis		histological analysis	
	explantation time	successful graft explantation	successful contralateral carotid explantation	graft	carotid	graft	carotid
Subject I	day 10	–	+	–	+	–	–
Subject II	day 10	+	+	–	+	+	+
Subject III	day 50	+	+	–	+	+	–
Subject IV	day 90	+	–	–	–	+	–

In Subject I, the contralateral carotid artery was carefully isolated and promptly delivered for mechanical evaluation (Figure 7a). However, due to the inability to explant the graft while preserving its structural integrity, no further pathological or mechanical analyses could be performed on this subject. In Subject II, the contralateral artery was similarly processed and mechanically tested. However, the graft specimen had become significantly stiffened (Figure 7b), which complicated dissection and prevented the acquisition of an intact sample suitable for mechanical testing; therefore, the graft was only subjected to histological analysis.

**Figure 7:**
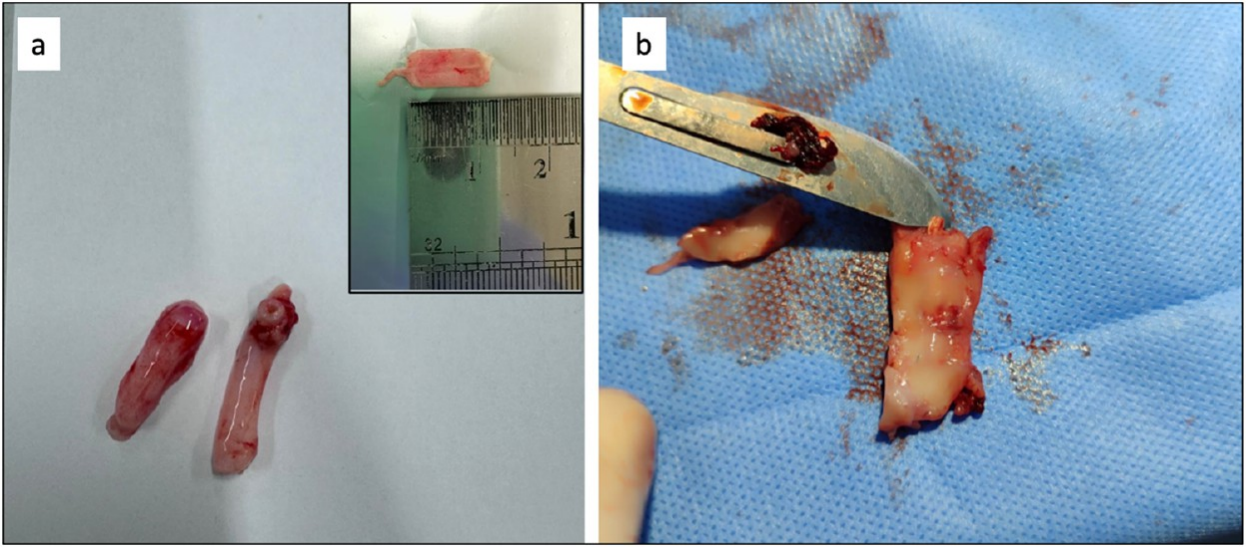
(a) Explanted contralateral carotid artery from Subject I (for mechanical analysis) and (b) Stiffen graft sample explanted from Subject II (for pathological examination).

In Subjects III and IV, sacrificed on days 50 and 90, respectively, both grafts and contralateral carotid arteries were successfully explanted with preserved structural integrity. The graft samples were fixed in formaldehyde along with adjacent tissue for histological analysis (Figure 8); however, they could not be subjected to mechanical testing due to the difficulty in separating them from surrounding tissues.

**Figure 8:**
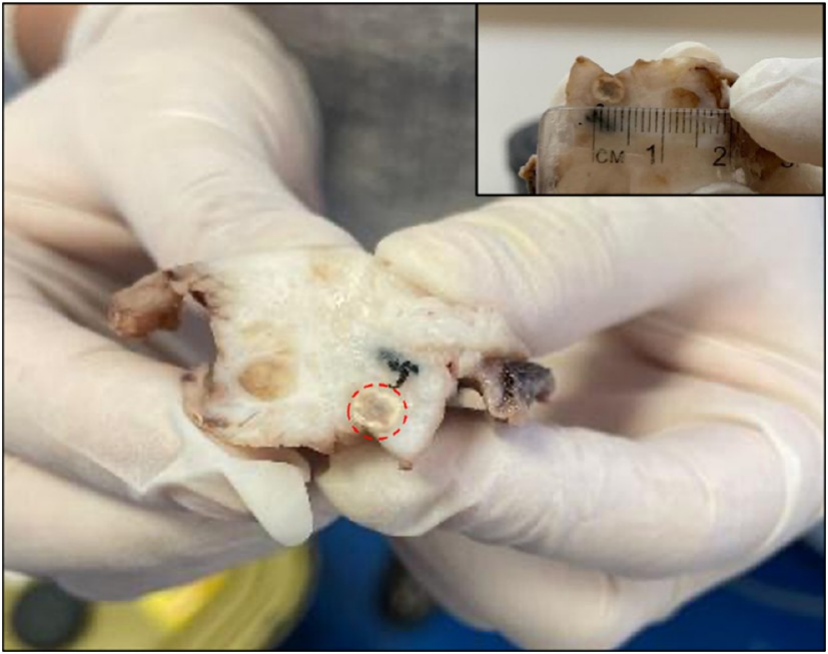
Graft samples explanted from Subject III preserved in formaldehyde and sectioned for histological analysis (highlighted by a red circle).

### Postmechanical Assessment

To evaluate the mechanical performance of the bilayer vascular grafts in comparison with porcine carotid arteries and to validate the tensile testing results for native arteries, contralateral carotid arteries explanted from Subjects I, II, and III were also subjected to uniaxial tensile testing under conditions identical to those applied to the graft samples. On the other hand, the carotid arteries from Subject IV could not be included in tensile testing due to difficulties in explantation and compromised structural integrity. The results are presented in Table 9. The tensile strength of coronary arteries reported in the literature is approximately 1.44 MPa,^70^ with elongation values ranging from 45 to 99%.^71^ As can be seen, the tensile strength obtained in this study is close to the literature values, while the elongation exceeded those reported. Nevertheless, the fabricated bilayer graft samples exhibited superior mechanical properties compared to contralateral carotid arteries and demonstrated potential for clinical application. However, explanted vascular grafts from the subjects could not undergo subsequent mechanical testing as intended due to hardening from thrombosis and incomplete separation from the surrounding tissue.

**Table 9:** Tensile Strength and Elongation Values of Porcine Contralateral Carotid Arteries from Subjects I, II, and III

	test direction	tensile strength (MPa)	elongation at break (%)
Subject I	axial (tubular)	1.60 ± 0.21	364.18 ± 70.98
Subject II	axial (tubular)	1.27 ± 0.24	231.67 ± 26.48
Subject III	axial (tubular)	0.90 ± 0.11	144.53 ± 13.89

### Histology

H&E staining images of the control group and vascular graft specimens from Subjects II, III, and IV are presented in Figure 9. In the PCLPLA_PLCL specimen obtained from Subject II, the vascular lumen appeared patent, the vessel wall thickness remained within normal limits, and the structural integrity was preserved. The endothelial layer was largely intact although mild edema was observed in the *tunica intima*. The elastic fibers exhibited a normal histological appearance. However, in subsequent sections, luminal narrowing and endothelial denudation were identified along with fibrin accumulation in the intimal layer. Signs of elastic fiber degeneration became more prominent, and the graft contour was visible in silhouette. Overall, PCLPLA_PLCL demonstrated a moderate tissue response to graft implantation, characterized by fibrin deposition and the partial loss of elastic fibers. Nevertheless, complete occlusion was not observed, and partial luminal patency was maintained.

**Figure 9:**
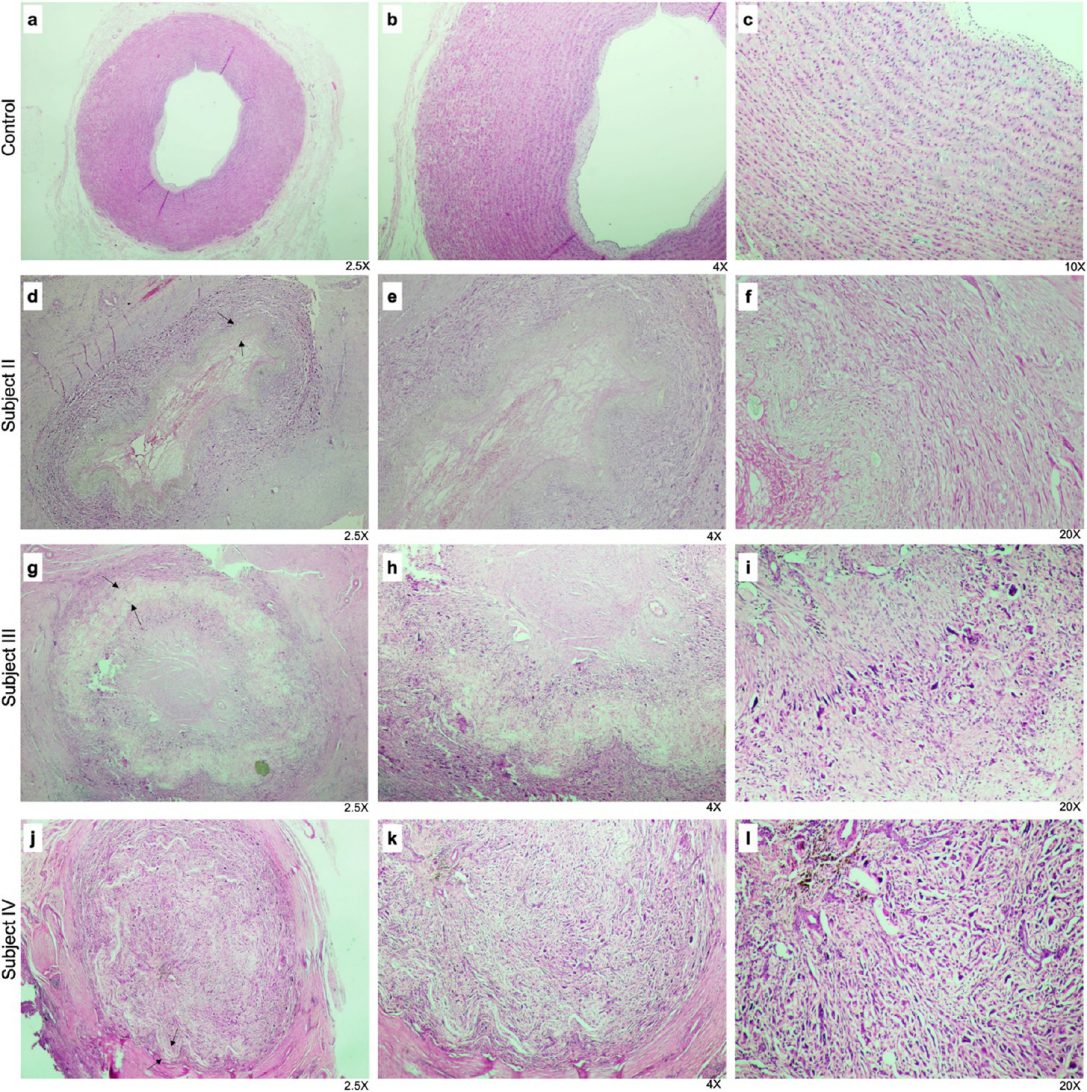
H&E staining images of the control group (a, b, c) and graft specimens from Subject II (PCLPLA_PLCL; d, e, f), Subject III (PCL_PCL; g, h ,i), and Subject IV (PCL_PCL; j, k, l). The graft site is indicated by arrows in figure sections (d, g, j).

In the PCL_PCL graft obtained from Subject III, more severe histopathological alterations were recorded. The vascular lumen appeared completely obliterated, with evidence of fibrin undergoing hyalinization and marked degeneration of the elastic layer. In addition, prominent foreign body-type multinucleated giant cells were observed surrounding the graft, indicating a pronounced foreign body reaction to the graft material.^72^ These findings suggested that the PCL_PCL graft was recognized by the host tissue as a foreign material, triggering a chronic inflammatory response that ultimately resulted in complete luminal obstruction and a loss of graft function.

The PCL_PCL graft retrieved from Subject IV exhibited histopathological features closely resembling those observed in the graft from Subject III. The vascular lumen was entirely occluded and filled with fibrohyalinized material. The graft contour was only faintly discernible, and the elastic layer was no longer visible. Prominent histopathological findings included extensive fibrosis, hyalinization, pronounced neovascularization, and the formation of foreign body-type inflammatory granulation tissue.

In addition to luminal changes, histological analysis revealed prominent fibroblastic and histiocytic cell infiltration at the graft periphery, with gradual and partial cellular penetration progressing from the outer surface toward the inner regions of the nanofibrous wall, while complete transmural infiltration was not observed at the studied time points.

Overall, the findings suggested that progressive inflammation, loss of the elastic layer, fibrin accumulation, and foreign body reaction were the primary mechanisms contributing to vascular occlusion following graft implantation.^73^ While the PCLPLA_PLCL graft obtained from Subject II reflected an earlier stage of the pathological process, the PCL_PCL grafts from Subjects III and IV exhibited a more advanced inflammatory response and predominant fibrotic alterations. This condition could be explained by the fact that the graft sample from Subject II was explanted on day 10, while the grafts from Subjects III and IV were explanted on days 50 and 90, respectively, when the vessels were almost completely occluded.

## Conclusion

This study presented the comprehensive development and evaluation of electrospun bilayer vascular grafts composed of PCL, PLA, and PLCL, integrating material characterization, mechanical testing, *in vitro* cell compatibility, and *in vivo* implantation in a porcine carotid artery model. The fabricated grafts exhibited homogeneous fiber morphology and structurally stable bilayer configurations, with mechanical properties exceeding those of native arteries. Notably, both graft designs demonstrated burst pressures well above clinical benchmarks, while PCL_PCL grafts exhibited superior compliance and tensile strength. *In vitro* assays confirmed the biocompatibility of all polymer compositions with endothelial and smooth muscle cells, with PCL/PLA-based surfaces notably enhancing HUVEC proliferation over time. These results highlighted the mechanical robustness and biological performance of the electrospun bilayer grafts, particularly those incorporating PCL.

The *in vivo* outcomes underscore the critical role of both the material design and pharmacological support in maintaining graft patency. PCLPLA_PLCL grafts experienced early occlusion, likely due to interfacial delamination and reduced compliance, whereas PCL_PCL grafts retained partial patency for up to 90 days in subjects receiving anticoagulant treatment. While these findings were promising, especially within the context of a large animal model, histological evaluations revealed fibrotic encapsulation and foreign body reactions in all explanted grafts at later stages, indicating the need for further optimization.

Taken together, these results emphasize that the successful development of small-diameter vascular grafts depends not only on mechanical performance or biocompatibility but also on achieving a fine-tuned balance between structural integrity, compliance, interfacial cohesion, pharmacological strategy, and host-specific biological responses. In this context, the combined attainment of clinically relevant burst resistance, physiological compliance, and sustained partial patency in a porcine carotid model aligns with several key preclinical benchmarks commonly considered for small-diameter vascular graft translation. The prolonged patency observed in one subject, together with the consistent performance of the PCL_PCL grafts, suggests a promising developmental trajectory. Further refinements, including the incorporation of sustained drug delivery strategies and improved surgical precision, will be essential to support the long-term success and clinical translation.
